# The Mechanosensory Lateral Line System Mediates Activation of Socially-Relevant Brain Regions during Territorial Interactions

**DOI:** 10.3389/fnbeh.2016.00093

**Published:** 2016-05-13

**Authors:** Julie M. Butler, Karen P. Maruska

**Affiliations:** Department of Biological Sciences, Louisiana State UniversityBaton Rouge, LA, USA

**Keywords:** assessment, cichlid, mechanoreception, sensory processing, social behavior, social decision-making, teleost

## Abstract

Animals use multiple senses during social interactions and must integrate this information in the brain to make context-dependent behavioral decisions. For fishes, the largest group of vertebrates, the mechanosensory lateral line system provides crucial hydrodynamic information for survival behaviors, but little is known about its function in social communication. Our previous work using the African cichlid fish, *Astatotilapia burtoni*, provided the first empirical evidence that fish use their lateral line system to detect water movements from conspecifics for mutual assessment and behavioral choices. It is unknown, however, where this socially-relevant mechanosensory information is processed in the brain to elicit adaptive behavioral responses. To examine for the first time in any fish species which brain regions receive contextual mechanosensory information, we quantified expression of the immediate early gene *cfos* as a proxy for neural activation in sensory and socially-relevant brain nuclei from lateral line-intact and -ablated fish following territorial interactions. Our *in situ* hybridization results indicate that in addition to known lateral line processing regions, socially-relevant mechanosensory information is processed in the ATn (ventromedial hypothalamus homolog), Dl (putative hippocampus homolog), and Vs (putative medial extended amygdala homolog). In addition, we identified a functional network within the conserved social decision-making network (SDMN) whose co-activity corresponds with mutual assessment and behavioral choice. Lateral line-intact and –ablated fight winners had different patterns of co-activity of these function networks and group identity could be determined solely by activation patterns, indicating the importance of mechanoreception to co-activity of the SDMN. These data show for the first time that the mechanosensory lateral line system provides relevant information to conserved decision-making centers of the brain during territorial interactions to mediate crucial behavioral choices such as whether or not to engage in a territorial fight. To our knowledge, this is also the first evidence of a subpallial nucleus receiving mechanosensory input, providing important information for elucidating homologies of decision-making circuits across vertebrates. These novel results highlight the importance of considering multimodal sensory input in mediating context-appropriate behaviors that will provide broad insights on the evolution of decision-making networks across all taxa.

## Introduction

Sensory signals from conspecifics provide relevant and necessary information for animals to make context-appropriate behavioral decisions. The social behavior network (SBN) is a group of conserved brain nuclei that possess sex steroid receptors, are reciprocally connected, and are involved in a variety of social behaviors, such as aggression, courtship, spawning, and parental care (Newman, 1999; Goodson, 2005). The SBN, in conjunction with the mesolimbic reward system, comprise the conserved social decision-making network (SDMN), which is thought to evaluate the salience of social cues to produce suitable behavioral responses (O'Connell and Hofmann, 2011). Many of the nuclei (or nodes) of the SDMN are connected with sensory processing regions but the relative role of inputs from different sensory modalities in mediating behavioral responses remains relatively unexplored, particularly in teleost fishes, the largest, and most diverse group of vertebrates.

The teleost mechanosensory lateral line system allows fish to detect near flow water movements (Dijkgraaf, 1963), such as those created by water currents, approaching predators, or conspecifics during social interactions (reviewed in Montgomery et al., 2014). Lateral line-mediated vibrational communication is used by several fish species during reproductive and spawning behaviors (Satou et al., 1994; Mirjany et al., 2011; Medina et al., 2013), and the African cichlid fish *Astatotilapia burtoni* uses hydrodynamic cues during territorial interactions (Butler and Maruska, 2015). Mechanosensory cues are intentionally produced by many fish species as a form of communication (Satou et al., 1994; Mirjany et al., 2011; Medina et al., 2013; Butler and Maruska, 2015), and without a functioning lateral line system, fish have altered behaviors and are less likely to engage in social interactions. For example, lateral line-ablated *A. burtoni* dominant males were ~50% less likely than lateral line-intact males to engage in a territorial interaction (Butler and Maruska, 2015), and lateral line-ablated hime salmon males were less likely to display courtship and spawning behaviors (Satou et al., 1994). In addition, the *A. burtoni* lateral line system is used to assess opponents and facilitates the use of less dangerous non-contact behaviors over contact behaviors when engaged in male-male territorial contests (Butler and Maruska, 2015). Despite the known importance of the lateral line system in mediating these reproductive and territorial interactions, it remains unknown where this socially-relevant mechanosensory information is processed in the brain to facilitate behavioral decisions.

The highly social African cichlid fish *A. burtoni* is an ideal model system to examine the role of hydrodynamic information in mediating activation of socially-relevant brain regions. *A. burtoni* have a diverse behavioral repertoire (Fernald, 1977; Fernald and Hirata, 1977) that produces a variety of multimodal sensory signals during social interactions (visual: Chen and Fernald, 2011, chemosensory: Maruska and Fernald, 2012, auditory: Maruska et al., 2012b, mechanosensory: Butler and Maruska, 2015). Males exist as two distinct phenotypes, dominant and subordinate, and can rapidly switch between the two depending on their social environment, with rapid consequences for their reproductive physiology (Maruska and Fernald, 2013; Maruska et al., 2013; Maruska, 2014). Dominant males are brightly-colored, actively court females, and defend their spawning territory from other males (Fernald, 1977; Fernald and Hirata, 1977), and many of these behaviors involve fin and body movements that produce hydrodynamic signals detected by the lateral line system of near-by fish. But where in the brain are these socially-relevant hydrodynamic signals processed to produce behavioral changes?

Although not previously investigated, it is possible that the lateral line system provides relevant information to the SDMN to influence behavioral output during social interactions. By quantifying brain activation using an immediate early gene (*cfos*) as a proxy for neural activation, we identify for the first time in any fish species which brain regions are important for processing socially-relevant mechanosensory information in a territorial context. We first examined individual nodes of the SDMN to investigate whether these regions received mechanosensory input, and then combined activation data to examine the role of mechanosensory information on functional connectivity and co-activation across the SDMN.

## Materials and methods

### Experimental animals

Adult *A. burtoni* were bred from a wild-caught stock from Lake Tanganyika, Africa, and were maintained in an environment that mimicked their natural habitat. *A. burtoni* were housed in 30L aquaria at 28–30°C on a 12L:12D cycle and fed cichlid flakes (AquaDine, Healdsburg, CA) once daily and supplemented with brine shrimp. All experiments were performed in accordance with the recommendations and guidelines stated in the National Institutes of Health (NIH) Guide for the Care and Use of Laboratory Animals, 2011. The protocol was approved by the Institutional Animal Care and Use Committee (IACUC) at Louisiana State University, Baton Rouge, LA. The animals used in this experiment are a subset of animals used in previously published behavioral analyses of the role of the mechanosensory lateral line system during male-male territorial interactions (Butler and Maruska, 2015).

### Behavior experiments

**Figure 1:**
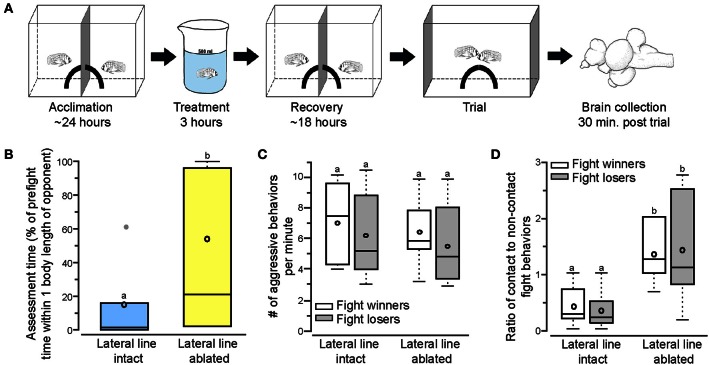
**Lateral line-ablated animals have decreased assessment abilities and prefer contact over non-contact fight behaviors. (A)** Two dominant *Astatotilapia burtoni* males acclimated on either side of an opaque blue barrier for 24 h with a quarter of a terracotta pot to serve as a territory. Fish were treated for 3 h (intact: normal cichlid-system water; ablated: 2mM cobalt chloride in low calcium cichlid-system water) and placed back in the experimental tank to recover for 18 h prior to behavioral testing. Total acclimation time, including treatment, was ~2 days. On the day of the trial, the center barrier was removed and the two quarter pots repositioned to form a single half-pot territory to induce a territorial fight. Opaque barriers were placed on both ends of the tank to block the view of neighboring fish during trials. Animals were sacrificed and brains collected 30 min after the fight concluded. Modified from Butler and Maruska (2015). **(B)** Lateral line-ablated fish (yellow) had increased assessment time (quantified as the percent of pre-fight time spent within one body length of the opponent without performing an aggressive behavior) than lateral line-intact fish (blue). **(C)** The number of aggressive behaviors per minute was not different between lateral line-intact and lateral line-ablated fight winners and losers. **(D)** Lateral line-ablated fish used more contact than non-contact fight behaviors (>1 signifies reliance on contact behaviors, < 1 signifies preference for non-contact behaviors). Different letters indicate statistical significance at *P* < 0.05. Tukey's box plots were used to represent behavior data (*N* = 6 per group): data median is represented by a line and data mean by an open circle, the box extends to the furthest data points within the 25th and 75th percentile, and whiskers extend to the furthest data points in the 25th/75th percentiles. Absence of whiskers indicates absence of data points outside of the 25th/75th percentiles. Data points outside the 25th/75th percentiles are represented by closed gray circles.

We previously demonstrated that the mechanosensory lateral line system in *A. burtoni* is used during territorial interactions (Butler and Maruska, 2015). Here, we used the same paradigm as previously described in which dominant male fish were forced to compete over a novel territory (Figure 1A). A single 10-gallon tank was divided into two equal compartments (25.4 × 31.2 × 25.4 cm) by a removable opaque acrylic barrier and a quarter terra-cotta pot was placed on either side of the barrier to serve as a territory for each subject fish. Experimental male fish were chosen based on their displays of typical dominance behaviors in community tanks for ~5 days before being moved to the experimental tank to acclimate for 2 days (including treatment time) in their new territory. Each animal was used for only one behavior trial. Animals were size-matched for both SL (41.000 ± 4.283 mm, mean ± SD) and BM (2.128 ± 1.828 g) so that no fish was more than 10% larger than his opponent, and to ensure that SL and BM did not differ between trial pairings (SL: Mann-Whitney Rank Sum: *U* = 69.000, *T* = 153.000, *P* = 0.884; BM: *U* = 45.000, *T* = 177.000, *P* = 0.126).

On the morning of the experimental trial, fish were allowed to acclimate to the video camera for ~10 min before the camera was turned on to record a 5-min pre-trial behavior baseline. The opaque barrier was then removed and the two quarter pots were repositioned to form a single territory shelter. In this paradigm, the two dominant males with individual territories are then forced to fight over the new single territory shelter in the same tank and have equal opportunity to acquire the territory. During the trial time, opaque barriers were placed on either end of the tank to block the view of other fish in adjacent tanks.

All trials were recorded and later quantified for prefight and fight behaviors, including contact (one fish physically touching the other) and non-contact (within one body length but not physically touching) behaviors. Non-contact behaviors included lateral displays (fish orient parallel or perpendicular to each other, erect fins, distend jaws, and shake their bodies), lunges (quick movement toward opponent without physical contact), and frontal threats (fish distends jaw and flares opercula while moving toward opponent). Contact behaviors included abnormal lateral displays (lateral display but with physical contact to the opponent), mouth fights (opponents grasp one another by the mouth and push, pull, and turn), bites (with mouth open, one fish makes physical contact with the opponent), and nudges/rams (with mouth closed, one fish pushes the other). Fight onset was defined as the first reciprocal exchange of aggressive behaviors, and fights had to last a minimum of 30 s. Fish were allowed to fight until a clear winner and loser was established based on the following criteria as outlined previously (Maruska et al., 2013; Butler and Maruska, 2015). The winner fish had to fulfill two of the following characteristics: (1) entered the shelter >3 times within a 1-min period, (2) entered and stayed in the shelter for >10 consecutive seconds, (3) performed at least 3 dominance behaviors within 1 min, and (4) chased or bit the other male. The subordinate, or loser, fish had to fulfill both of the following criteria: (1) loss of eye bar and bright coloration, and (2) perform typical submissive behaviors (e.g., fleeing, hiding at the top of the water column). Latency to fight was defined as the time between removing the barrier and the fight onset, and fight duration as the time between fight onset and establishment of a winner. Assessment time was defined as the percent of prefight time spent within one body length of the opponent without performing an aggressive display.

### Mechanosensory lateral line ablation

To compare behavior of lateral line-intact and lateral line-ablated fish, experimental fish were randomly assigned to one of two groups prior to use in behavioral experiments described above: sham treatment or lateral line ablation. Lateral line ablation was done by immersing fish in cichlid-system fish water containing 2 mM cobalt chloride hexahydrate (CoCl_2_; Sigma) and 1 mM EGTA (ethylene glycolbis tetraacetic acid; Sigma-Aldrich) for 3 h. Immediately following CoCl_2_ treatment, fish were placed in ice water for ~3 min before the posterior lateral line nerve was bilaterally transected. To cut the nerve, 2–3 scales were gently removed at the posterior dorsal edge of the operculum and a #11 scalpel was used to make a small incision through the skin and superficial muscle about 4 mm in length. The posterior lateral line nerve (pLLn) was then visible and a 1–2 mm portion of the nerve was removed. Using a dose of 2 mM CoCl_2_ for 3 h consistently ablated neuromasts located on the head. This treatment, however, left neuromasts on the trunk and tail of the animals intact. To resolve this issue, we bilaterally clipped the pLLns to remove mechanosensory input from the remaining intact neuromasts on the trunk and tail. Together, these treatments removed lateral line input from both the cranium (due to CoCl_2_-treatment) and the trunk (pLLn transections). The efficacy of this treatment was previously established, and our previous study demonstrated no toxicity effects from the cobalt chloride treatment (Butler and Maruska, 2015).

Sham handled fish (i.e., lateral line-intact fish) were transferred to a similar-sized container as the ablated fish and immersed in normal cichlid-system fish water for 3 h. Following treatment, fish were placed in ice water and a small incision was made into the dorsal musculature near the dorsal fin to insure that it did not affect the lateral line system. Sham treatment was used to control for handling stress (e.g., 3 h in beaker and minor surgery). Following treatment or sham-handling, fish were returned to their half of the experimental tank overnight, and behavior experiments took place 18 h after the end of treatment, a time during which neuromast function in the cobalt-treated fish did not recover (verified with DASPEI staining).

### Tissue collection

To compare brain activation patterns of lateral line-intact and -ablated fish, we collected brains from six intact-intact pairs (i.e., six winners, six losers) and six ablated-ablated pairs. Fish were quickly removed from the experimental tank 30-min post fight (using the above criteria), anesthetized in ice cold fish water, measured for standard length (SL) and body mass (BM), and killed by rapid cervical transection. Brains were exposed and fixed in the head overnight at 4°C in 4% paraformaldehyde (PFA) in 1 × phosphate-buffered saline (1 × PBS), rinsed for 24 h in 1 × PBS, and cryoprotected overnight in 30% sucrose in 1 × PBS. Gonads were removed and weighed (gonad mass, GM) to calculate gonadosomatic index [GSI = (GM/BM) ^*^ 100; 0.846 ± 0.173]. GSI did not differ among treatment groups (*t* = −0.083; *df* = 22; *P* = 0.934). Brains were then embedded in OCT media (TissueTek, Sakura), sectioned in the transverse plane on a cryostat (Leica, CM1850) at 20 μm, and collected onto 2 alternate sets of charged slides (VWR superfrost plus). Slides were dried flat at room temperature for 2 days prior to storage at −80°C.

### Preparation of dig-labeled riboprobe for *in situ* hybridization

The immediate early gene *cfos* is commonly used as a marker of neural activation. To visualize *cfos* mRNA in the brain, we used chromogenic *in situ* hybridization (ISH) with a riboprobe specific to the *A. burtoni cfos* mRNA sequence. Primers were designed based on the sequence available in Genbank (HQ232413.1) and commercially synthesized (Life Technologies; forward primer: 5′-agagaactgatcgggagcagcgct-3′; reverse primer: 5′-caggttgggatatcattctgcagg-3′). Probe template was generated by PCR amplification (Platinum SuperMix, Life Technologies) of whole brain *A. burtoni* cDNA, *cfos* gene-specific primers, and the following reaction conditions: 95°C for 1 min, 45 cycles of: (95°C for 15 s, 55°C for 15 s, 72°C for 1 min), and 72°C for 1 min. A transcription reaction was used to incorporate DIG (DIG-labeling mix, Roche)-labeled nucleotides into the purified PCR template (MinElute PCR Kit, Qiagen) before probe purification (GE Illustra Probe Quant G-50 microcolumns). PCR products and the final probe were checked on a 1% agarose gel after each step and verified as a band of the correct size. The probe was then diluted 1:5 in hybridization buffer and stored at −20°C until use. Probes were transcribed using the T3 polymerase transcription initiation sequence (aattaaccctcactaaaggg) that was added to the reverse (for anti-sense probes) or forward (for sense control probes) primer.

### *In situ* hybridization

*In situ* hybridization was performed to visualize and quantify brain activation differences between lateral line-intact and -ablated fish following territorial interactions. To minimize inter-ISH variability for quantification, each staining reaction contained brains from individuals from each treatment group. Slides of cryosectioned brains were thawed to room temperature and a hydrophobic barrier (Immedge pen, Vector Laboratories) was applied around the sections and allowed to dry for 40 min. Slides were incubated at room temperature in 1 × PBS (3 × 5 min), 4% PFA (20 min), 1 × PBS (2 × 5 min), proteinase K (10 μg/ml final conc. in 50 mM Tris-HCl pH 7.5, 5 mM EDTA pH 8.0; 10 min), 1 × PBS (10 min), 4% PFA (15 min), 1 × PBS (2 × 5 min), milliQ water (3 min), 0.25% pure acetic anhydride in 0.1 M triethanolamine-HCl pH 8.0 (10 min), and 1 × PBS (5 min). Slides were then placed in a sealed humidified chamber at 60–65°C and incubated in pre-warmed hybridization buffer without probe (50% formamide, 5 × SSC, 0.1% tween-20, 0.1% CHAPS, 5 mM EDTA, 1 mg/ml torula RNA) for 3 h. Slides were incubated with *cfos* riboprobe, sealed with hybrislips (Life Technologies) to evenly distribute probe and prevent drying, and placed in a sealed, humidified chamber in a hybridization oven at 60–65°C. After 12–16 h of hybridization, hyrbislips were removed in pre-warmed 2 × SSC:50% formamide with 0.1% tween-20 solution and the following washes were performed at 60–65°C: 2 × SSC:formamide (2 × 30 min), 2 × SSC:Maleate Buffer (MABT; 100 mM maleic acid pH 7.2, 150 mM NaCl, 0.1% tween-20; 2 × 15 min), and MABT (2 × 10 min). Slides were then washed at room temperature in MABT (2 × 10 min), blocked in MABT with 2% bovine serum albumin (3 h) at room temperature, and incubated in alkaline-phosphatase-conjugated anti-DIG Fab fragments (Roche; 1:5000 dilution in blocking solution) overnight (12–16 h) at 4°C in a humidified chamber. Slides were then rinsed in MABT at room temperature (3 × 30 min), incubated in alkaline phosphate buffer (2 × 5 min), and developed with nitro-blue tetrazolium/5-bromo-4-chloro-3′-indolyphosphate (NBT/BCIP) substrate (Roche) at 37°C in the dark for ~3 h. Slides were then rinsed in 1 × PBS (3 × 5 min), fixed in 4% PFA (10 min), washed with 1 × PBS (3 × 5 min), and coverslipped with aquamount media (Thermo-Scientific).

**Figure 2:**
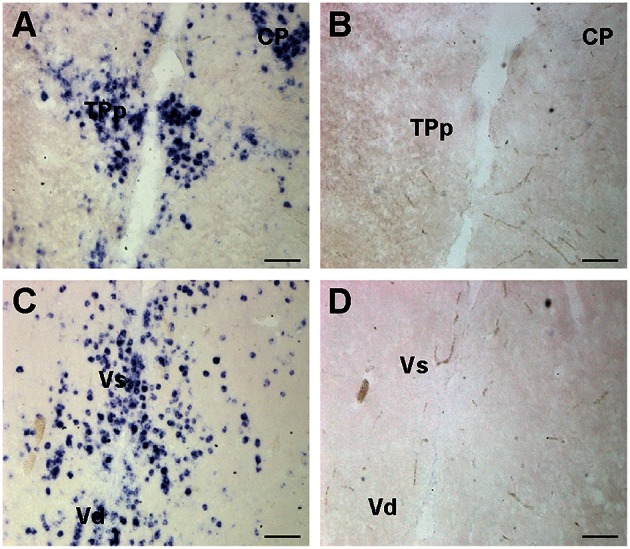
**Representative examples of *cfos in situ* hybridization staining to show probe specificity**. Photomicrographs of adjacent transverse sections from the same brain stained with *cfos* antisense **(A,C)** and sense control probes **(B,D)**. Sense control probes showed no staining in any region. Scale bars represent 100 μm. See list for abbreviations.

To test for probe specificity, additional brains were collected and prepared as above. A *cfos* sense control riboprobe was generated by adding the T3 sequence to the forward primer instead of the reverse primer (as in anti-sense probes). One set of slides was stained with sense probe while the alternate set was stained with anti-sense probe and run simultaneously. The sense controls did not show any labeling in the brain (Figure 2).

### Quantification of brain activation

To quantify differences in *cfos* staining, slides were visualized on a Nikon Eclipse Ni microscope and photographs were taken with a color digital camera controlled by Nikon Elements software. Brightfield and phase contrast were used to visualize neuroanatomical markers and brain nuclei in relation to DIG-labeled cells. A cresyl violet stained *A. burtoni* reference brain, *A. burtoni* brain atlas, and other relevant papers (Fernald and Shelton, 1985; Munchrath and Hofmann, 2010; Maruska et al., 2012a) were used for identification of neuroanatomical markers. Images were taken at the highest magnification that encompassed the entire area of interest. The following brain nuclei were analyzed: sensory regions: medial octavolateralis nucleus (MON), ventrolateral portion of the torus semicircularis (TSvl), central portion of the torus semicircularis (TSc; torus semicircularis = putative homolog of the mammalian inferior colliculus), central posterior thalamic nucleus (CP), posterior part of the dorsal telencephalon (Dp; putative homolog of mammalian piriform cortex); SDMN regions: anterior tuberal nucleus (ATn; homolog of ventromedial hypothalamus), central part of the dorsal telencephalon, subdivision 4 (Dc-4), granular zone of the lateral zone of the dorsal telencephalon (Dlg; homolog of medial pallium; hippocampus), magnocellular preoptic nucleus magnocellular division (nMMp), magnocellular preoptic nuclus parvocellular division (nPMp), periventricular nucleus of the posterior tuberculum (TPp; homolog of ventral tegemental area), caudal subdivision of the dorsal part of the ventral telencephalon (Vdc; homologous in part to nucleus accumbens/basal ganglia), supracomissural nucleus of the ventral telencephalon (Vs; homolog of central/medial/extended amygdala), ventral portion of the ventral telencephalon (Vv; homolog of lateral septum). The medial portion of the dorsal telencephalon (Dm; part of SDMN; homologous in part to pallial amygdala) was not quantified due to low *cfos* staining in all animals and the inability to reliably distinguish between its subdivisions. Similarly, the periaqueductal gray (PAG; part of SDMN) was not examined because of its small size and inability to distinguish it from surrounding nuclei. The lateral preglomerular nucleus (PGl; receives lateral line input) was also not quantified due to hazy staining instead of discrete cell staining and the inability to distinguish between mechanosensory and other sensory processing subdivisions. Other SDMN nuclei (Vc, central part of the ventral telencephalon [striatum]; VTn, ventral tuberal nucleus [anterior hypothalamus], and Vl, lateral part of the ventral telencephalon) were not quantified for similar reasons.

For nuclei with relatively low *cfos*-stained cell densities (e.g., MON), the region was outlined and the area was measured in Nikon Elements. Individual cells were then counted and cell density (cells/μm^2^) was calculated by dividing the number of cells by the total quantified nucleus area (μm^2^). For nuclei with high-density *cfos*-stained cells (e.g., ATn, CP), the region was outlined and gridlines (either 15 × 15 μm, or 50 × 50 μm dependent on magnification) were applied to the image. Numbers were assigned to all boxes that were fully encompassed by the outline and a random number generator was used to designate which boxes were used to count cell numbers. Three or five boxes per section were quantified depending on size of the region. Cell density was calculated by dividing the number of cells within the box by the area of the box. For both methods, four consecutive sections were quantified for each region and averaged together for a cell density value of that region in a particular animal. Cell quantification data were then averaged across all individual fish within each group. The entirety of each region was not quantified for every nucleus. All *cfos* quantification was done blind to fish identity.

### Anosmic controls

During analysis of *cfos* activation in the brain, we noted drastically reduced staining in olfactory processing regions such as the inner cellular layer of the olfactory bulb and the posterior portion of the dorsal telencephalon (Dp) in lateral line-ablated fish, suggesting that 2 mM CoCl_2_ treatment may also impair chemosensory systems. To ensure that any behavior differences were not due to lack of olfaction, we also included anosmic fish as a control in addition to the sham treated controls described above. To create anosmic fish, we used a micro-cauterizer (Cautery High Temp Adjust-A-Temp Fine Tip, Bovie Medical Corporation) to burn off and ablate the olfactory epithelium prior to placing fish in the same experimental paradigm used for our mechanosensory trials (*N* = 5). Fish were allowed to recover for 2 days prior to the forced territorial interaction. Brains from fish in three anosmic-anosmic trials were collected, stained for *cfos* mRNA, and analyzed as described above.

### Statistical analysis

Comparisons of GSI, SL, BM and behavior data with only one variable (i.e., intact vs. ablated) were compared with student's *t*-tests in SigmaPlot 12.3. If assumptions could not be met by transforming the data, non-parametric statistics were used and are reported appropriately throughout the text. Because winners and losers came from the same interactions and cannot be considered independent, linear mixed models were used to examine the impact of treatment (intact vs. ablated vs. anosmic) and outcome (winner vs. loser) on behavior data and brain activation data using SPSS 19. Two random effects were used, one for individual subjects and the other for each dyad. Pairwise comparisons with least significant difference (LSD) adjustments were used to determine differences between treatment (intact vs. ablated vs. anosmic). Pearson correlations were used to test for correlations between *cfos* staining data of each brain nucleus and other regions and behaviors. Corrections for multiple testing were done by controlling for the false discovery rate (FDR) using the Benjamini-Hochberg procedure with an FDR of 0.25. All significant *p*-values remained significant after FDR corrections for FDR, so only the exact *p*-values and effect size (e.g., *r*-values) are included in each table. We chose not to use Bonferroni corrections or other similar procedures because they reduce statistical power and increase the chance of type II errors, especially in small sample sizes. While these tests do reduce type I errors, their unacceptable effects on statistical power can hide potential biologically relevant results (Nakagawa, 2004). Unless otherwise stated, all animals were combined for correlations and multivariate analysis. Factor analyses were performed in SPSS using principal component extractions with Eigenvalues >1. Missing values were excluded listwise and small coefficients (<0.3) were suppressed. Components 1 and 2 were plotted in rotated space (varimax rotation). Only regions of the SDMN and mechanosensory processing regions were included in the multivariate analyses (TSc, Dp, Dc-4 removed). Similarly, only SDMN and mechanosensory processing regions were used in the discriminant function analysis, and missing values were replaced with the group mean for the discriminant function analysis only. All groups were considered equal during the classification, and classification was done using within-group covariance.

## Results

### Lateral line ablation and impact on social behavior

To examine territorial behaviors of lateral line-intact and -ablated animals we used a previously described behavioral paradigm in which two dominant *A. burtoni* males have equal opportunity to acquire a novel territory shelter (Figure 1A; Butler and Maruska, 2015). Territorial interactions occurred between two lateral line-intact males (*N* = 6 trials; 12 fish) or two lateral line-ablated males (*N* = 6 trials; 12 fish) and were scored for stereotypical aggressive and assessment behaviors. Lateral line-ablated fish spent significantly more time within one body length of their opponent (Figure 1B; *t*-test; *t* = −3.268, *df* = 10, *P* = 0.008), suggesting a decrease in assessment abilities similar to that previously described (Butler and Maruska, 2015). Latency to fight and fight duration, however, were similar in trials between two lateral line-intact and two lateral line-ablated fish (latency: *t* = −0.711, *df* = 10, *P* = 0.493; fight time: *t* = 1.174, *df* = 10, *P* = 0.268). Although aggressive scores (number of aggressive behaviors per minute) were similar for all fish (Figure 1C; LMM; treatment: *F* = 0.598, *P* = 0.453; outcome: *F* = 0.700, *P* = 0.421; treatment × outcome: *F* = 0.015, *P* = 0.905), lateral line-ablated males relied more on contact aggressive behaviors while lateral line-intact males used predominantly non-contact aggressive behaviors (Figure 1D; treatment: *F* = 11.766, *P* = 0.005; outcome: *F* = 0.279, *P* = 0.611; treatment × outcome: *F* = 0.017, *P* = 0.900). These data indicate that the lateral line system is involved in opponent assessment and the facilitation of non-contact behaviors, as previously reported in *A. burtoni* (Butler and Maruska, 2015).

### Activation of sensory processing brain regions

**Figure 3:**
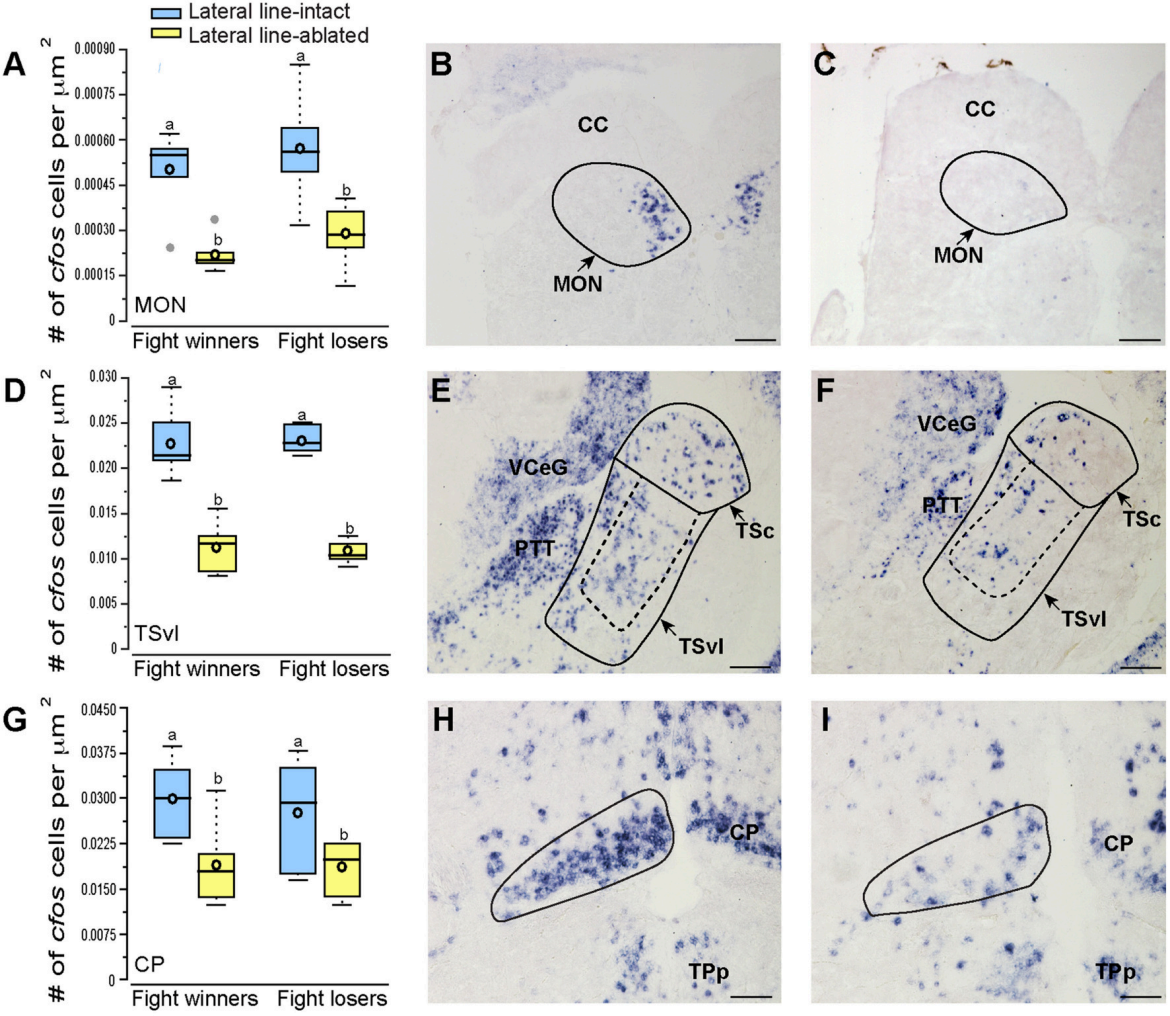
**Lateral line-ablated animals have decreased *cfos* staining in lateral line processing regions of the brain**. Quantification of *cfos* staining in the MON **(A)**, TSvl **(D)**, and CP **(G)** in lateral line-intact (blue) and -ablated (yellow) fight winners and losers (*N* = 6 for all groups). Photomicrographs of representative *cfos* staining (dark blue label) in transverse sections through the MON of a lateral line-intact **(B)** and -ablated **(C)** animal, the TSvl of an intact **(E)** and ablated **(F)** animal, and the CP of an intact **(H)** and ablated **(I)** animal. Solid circles depict nuclei borders and dashed outlines represent the subarea quantified for each nucleus. If no dashed outline is present, the entire nucleus was quantified. Different letters indicate statistical significance at *P* < 0.05 using *post-hoc* LSD tests. Scale bars in **(B,C,E,F)** represent 100 μm. Scale bars in **(H,I)** represent 25 μm. See Figure 1 legend for box plot descriptions. See list for abbreviations.

Brains from lateral line-intact and -ablated fight winners and losers were collected following territorial interactions to compare *cfos* expression in nuclei implicated in sensory processing and social decision-making. Mechanosensory cues are detected by neuromasts, the functional units of the lateral line system (Dijkgraaf, 1963), and this hydrodynamic information is sent via the anterior and posterior lateral line nerves into the MON of the hindbrain (McCormick, 1983; Meredith, 1984; Wullimann and Grothe, 2014). Lateral line-ablated animals had decreased *cfos* staining in the MON compared to lateral line-intact animals (Figures 3A–C; LMM; treatment: *F* = 24.397, *P* < 0.001; outcome: *F* = 1.488, *P* = 0.234; treatment × outcome: *F* < 0.001, *P* = 0.996; *post-hoc* LSD: *P* < 0.001) indicating that our ablation method was successful. The MON sends ascending projections to the ventrolateral portion of the midbrain torus semicircularis (TSvl; Luiten, 1975; McCormick, 1982, 1989; Echteler, 1984). The TSvl processes lateral line cues while the central portion of the torus semicircularis (TSc) processes auditory signals. Lateral line-ablated animals had decreased *cfos* staining in the TSvl (Figures 3D–F; treatment: *F* = 42.093, *P* < 0.001; outcome: *F* = 0.210, *P* = 0.656; treatment × outcome: *F* = 0.125, *P* = 0.731; LSD: *P* < 0.001) but not the TSc (treatment: *F* = 0.113, *P* = 0.893; outcome: *F* = 1.731, *P* = 0.221; treatment × outcome: *F* = 0.001, *P* = 0.953) indicating that treatment had no impact on audition but further demonstrates impaired mechanoreceptive capabilities. In addition, lateral line-ablated animals had decreased staining in the central posterior thalamic nucleus (CP; Figures 3G–I; treatment: *F* = 7.374, *P* = 0.006; outcome: *F* = 0.259, *P* = 0.619; treatment × outcome: *F* = 0.106, *P* = 0.751; LSD: *P* = 0.003), which acts as a relay station for both auditory and mechanosensory signals (Finger and Tong, 1984). Since the TSc was not different between intact and ablated animals, the difference in activation of the CP is likely due to decreased mechanosensory processing in lateral line-ablated fish.

### Activation of socially-relevant brain regions

**Figure 4:**
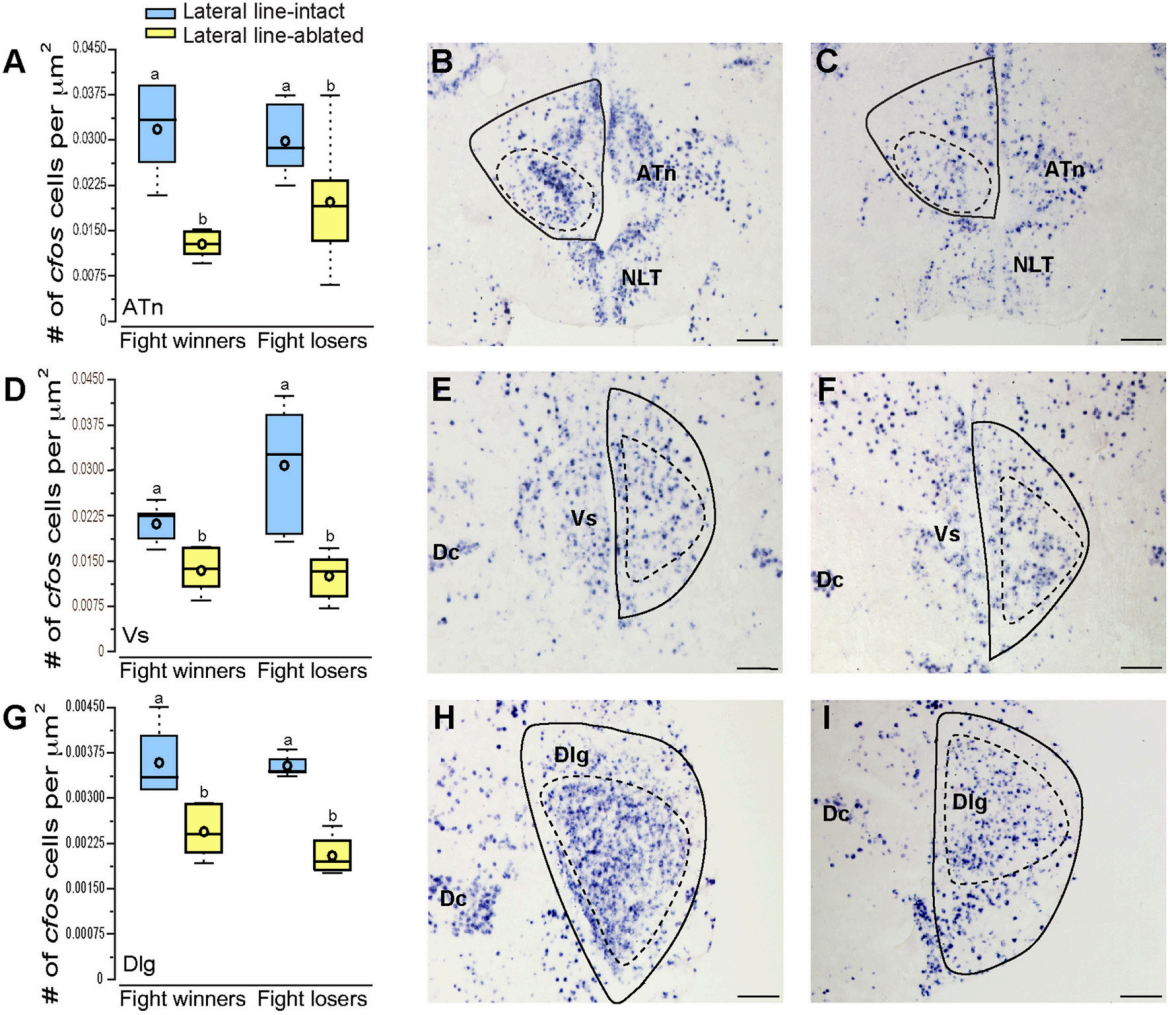
**Lateral line-ablated animals have decreased *cfos* staining in several SDMN nuclei**. Quantification of *cfos* staining in the ATn **(A)**, Vs **(D)**, and Dlg **(G)** in lateral line-intact (blue) and -ablated (yellow) fight winners and losers (*N* = 6 for all groups). Photomicrographs of representative *cfos* staining (dark blue label) in transverse sections through the ATn of a lateral line-intact **(B)** and -ablated **(C)** animal, the Vs of an intact **(E)** and ablated **(F)** animal, and the Dlg of an intact **(H)** and ablated **(I)** animal. Solid circles depict nuclei borders and dashed outlines represent the subarea quantified for each nucleus. Different letters indicate statistical significance at *P* < 0.05 using *post-hoc* LSD tests. Scale bars in **(B,C,E,F,H, I)** represent 100 μm. See Figure 1 legend for box plot descriptions. See list for abbreviations.

**Figure 5:**
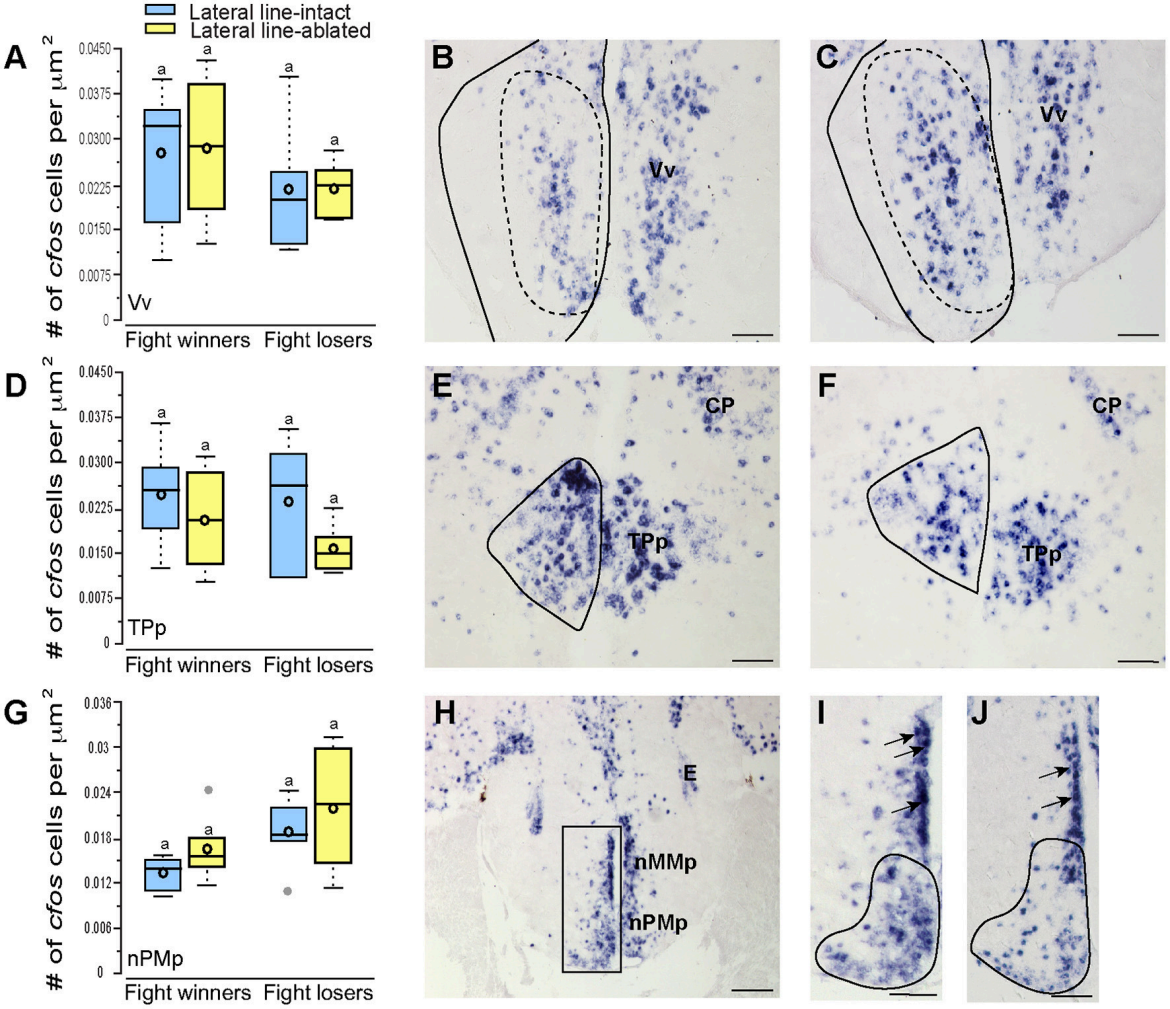
**Fight losers have increased activation of the nPMp but other SDMN regions are unaffected**. Quantification of *cfos* staining in the Vv **(A)**, TPp **(D)**, and nPMp **(G)** in lateral line-intact (blue) and -ablated (yellow) fight winners and losers (*N* = 5–6 for all groups). Photomicrographs of representative *cfos* staining (dark blue label) in the Vv of a lateral line-intact **(B)** and -ablated **(C)** animal, and the TPp of an intact **(E)** and ablated **(F)** animal. The preoptic area was divided into two parts, the nMMp, and the nPMp for quantification **(H)**. Representative photomicrographs in the POA of lateral line-intact fight winner **(I)** and loser **(J)**. Arrows in **(I,J)** indicate magnocellular cells quantified as part of the nMMp. Solid circles depict nuclei borders and dashed outlines represent the subarea quantified for each nucleus. If no dashed outline is present, the entire nucleus was quantified. Different letters indicate statistical significance at *P* < 0.05 using *post-hoc* LSD tests. Scale bars in **(B,C,E,F)** represent 25 μm. Scale bars in **(H)** represents 100 μm and scales in **(I,J)** represent 12.5 μm. See Figure 1 legend for box plot descriptions. See list for abbreviations.

To examine which brain regions process socially-relevant hydrodynamic cues, we quantified *cfos* expression in regions of the SDMN. Lateral line-ablated fish had decreased staining in the anterior tuberal nucleus (ATn; Figures 4A–C; treatment: *F* = 13.511, *P* < 0.001; outcome: *F* = 1.758, *P* = 0.212; treatment × outcome: *F* = 3.119, *P* = 0.106; LSD: *P* < 0.001), supracommissural nucleus of the ventral telencephalon (Vs; Figures 4D–F; treatment: *F* = 5.852, *P* = 0.010; outcome: *F* = 1.581, *P* = 0.226; treatment × outcome: *F* = 0.682, *P* = 0.421; LSD: P0.004), and the granular zone of the lateral zone of the dorsal telencephalon (Dlg; Figures 4G–I; treatment: *F* = 12.115, *P* < 0.001; outcome: *F* = 0.176, *P* = 0.679; treatment × outcome: *F* = 0.140, *P* = 0.713; LSD: *P* = 0.001), but none were affected by fight outcome (i.e., winning or losing). In contrast, lateral line-intact and -ablated animals had similar *cfos* staining in the ventral part of the ventral telencephalon (Vv; Figures 5A–C; treatment: *F* = 1.213, *P* = 0.33; outcome: *F* = 2.121, *P* = 0.176; treatment × outcome: *F* = 0.004, *P* = 0.948), the periventricular nucleus of the posterior tuberculum (TPp; Figures 5D–E; treatment: *F* = 1.493, *P* = 0.256; outcome: *F* = 1.036, *P* = 0.330; treatment × outcome: *F* = 0.546, *P* = 0.475), and the caudal subdivision of the dorsal part of the ventral telencephalon (Vdc; treatment: *F* = 0.272, *F* = 0.765; outcome: *F* = 1.187, *P* = 0.289; treatment × outcome: *F* = 0.046, *P* = 0.823). We quantified two different parts of the preoptic area (POA), the nMMp (magnocellular preoptic nucleus, magnocellular division) and the nPMp (magnocellular preoptic nucleus, parvocellular division). There was no effect of treatment or fight outcome on *cfos* staining in the nMMp (treatment: *F* = 0.566, *P* = 0.578; outcome: *F* = 1.610, *P* = 0.233; treatment × outcome: *F* = 1.396, *P* = 0.264) or the nPMp (Figures 5G–J; treatment: *F* = 0.062, *P* = 0.940; outcome: *F* = 1.119, *P* = 0.316; treatment × outcome: *F* = 0.400, *P* = 0.537). These data indicate that parts of the SDMN (ATn, Vs, Dlg) likely receive and process socially-relevant hydrodynamic cues while activation of other nuclei is independent of mechanosensory input, at least in this aggressive territorial context.

### Activation of brain regions in anosmic controls

**Figure 6:**
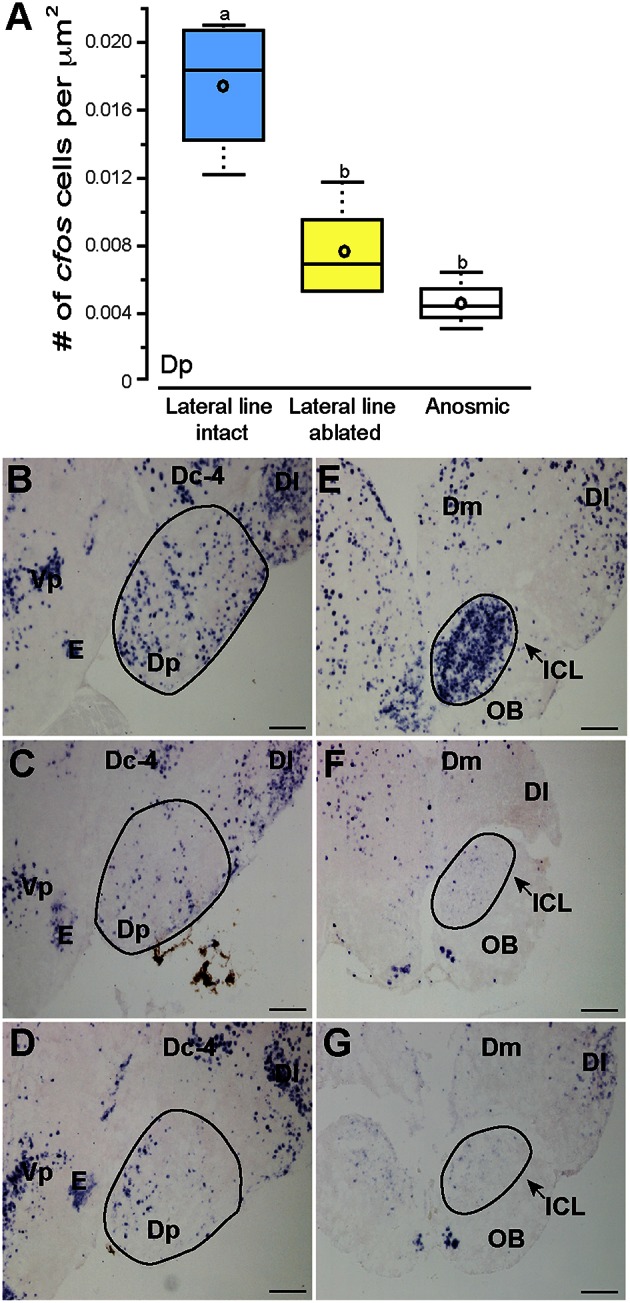
**Cobalt chloride treatment impaired olfactory processing in lateral line-ablated fish**. Quantification of *cfos* staining in the known telencephalic olfactory processing region, Dp **(A)** of lateral line-intact (blue; *N* = 6), lateral line-ablated (yellow; *N* = 5), and anosmic (white; *N* = 3) fight winners and corresponding photomicrographs of representative *cfos* staining in the Dp of a lateral line-intact **(B)** -ablated **(C)**, and anosmic **(D)** animal, and the olfactory bulbs of an intact **(E)**, ablated **(F)**, and anosmic **(G)** animal. Circled regions represent the area quantified for each nucleus. Different letters indicate statistical significance at *P* < 0.05 using *post-hoc* LSD tests. Scale bars in **(B–G)** represent 100 μm. See Figure 1 legend for box plot descriptions. See list for abbreviations.

Noticeably reduced DASPEI (vital dye used to verify lateral line ablation) staining of the olfactory epithelium following lateral line ablation suggested that cobalt chloride treatment also impaired olfaction (Butler and Maruska, 2015). To examine this, we compared *cfos* staining in the posterior portion of the dorsal telencephalon (Dp), a region known to process olfactory information in fishes (Satou, 1990) (Figure 6). Lateral line-ablated animals had reduced staining in the Dp (treatment: *F* = 4.992, *P* = 0.024; outcome: *F* = 0.056, *P* = 0.816; treatment × outcome: *F* = 0.949, *P* = 0.349; LSD: *P* = 0.012), but lateral line-ablated and anosmic fish have similar *cfos* staining in the Dp (LSD: *P* = 0.570). Thus, we included anosmic (i.e., olfactory epithelium ablated) animals in behavior trials and compared *cfos* staining of anosmic fight winners to lateral line-intact fight winners to ensure that any observed differences were due to impairment of the lateral line system and not comorbid effects on chemosensory systems.

Lateral line-intact and anosmic fight winners had similar *cfos* staining in all previously mentioned brain regions (MON, TSvl, TSc, CP, ATn, Vs, Dlg, Vv, TPp, Vdc, nMMp, and nPMp; *P* > 0.05 for all, see Table 1 for statistics), suggesting that impaired olfaction did not contribute to *cfos* staining differences in these regions between lateral line-intact and -ablated fish. Lateral line-ablated and anosmic animals had higher *cfos* staining in the central part of the dorsal telencephalon, subdivision 4 (Dc-4; treatment: *F* = 3.609, *P* = 0.050; outcome: *F* = 1.039, *P* = 0.336; treatment × outcome: *F* = 0.002, *P* = 0.964; LSD: *P* = 0.009) compared to lateral-line-intact fish. Because this difference is partly a result of impaired olfaction, Dc-4 was removed from multivariate analyses.

Table 1**Effect of treatment and outcome on *cfos* expression in sensory and socially-relevant brain regions**.

**Table:** 

	**Treatment**		**Outcome**		**Treatment x Outcome**	
	***F***	***P***	***F***	***P***	***F***	***P***
MON	24.397	**<0.001**	1.488	0.234	<0.001	0.996
TSvl	42.093	**<0.001**	0.210	0.656	0.125	0.731
CP	7.374	**0.006**	0.259	0.619	0.106	0.750
ATn	13.511	**<0.001**	1.758	0.212	3.119	0.106
Vs	5.852	**0.010**	1.581	0.226	0.682	0.421
Dlg	12.115	**<0.001**	0.176	0.679	0.140	0.713
TPp	1.493	0.256	1.036	0.330	0.546	0.475
nMMp	0.566	0.578	1.610	0.233	1.396	0.264
nPMp	0.062	0.940	1.119	0.316	0.400	0.537
Vv	1.213	0.333	2.121	0.176	0.004	0.948
Vdc	0.272	0.765	1.187	0.289	0.046	0.823
TSc	0.113	0.893	1.731	0.221	0.001	0.953
Dp	4.992	**0.024**	0.056	0.816	0.949	0.349
Dc−4	3.609	**0.050**	1.039	0.336	0.002	0.964

**Table:** 

**Multiple comparisons (treatment)**			
	**Sham-Ablated**	**Sham-Anosmic**	**Ablated-Anosmic**
	***P***	***P***	***P***
MON	**<0.001**	0.950	**<0.001**
TSvl	**<0.001**	0.781	**0.001**
CP	**0.003**	0.884	**0.023**
ATn	**<0.001**	0.722	**0.002**
Vs	**0.004**	0.812	**0.018**
Dlg	**0.001**	0.482	**0.002**
TPp	0.109	0.923	0.308
nMMp	0.543	0.571	0.923
nPMp	0.742	0.557	0.380
Vv	0.908	0.266	0.242
Vdc	0.491	0.759	0.909
TSc	0.947	0.908	0.873
Dp	**0.012**	0.059	0.570
Dc-4	0.129	**0.009**	0.133

*Main effects, interactions, and multiple comparisons were calculated using linear mixed models. Bold indicates significance at P < 0.05*.

### Correlations and multivariate analyses of multiple brain regions

**Figure 7:**
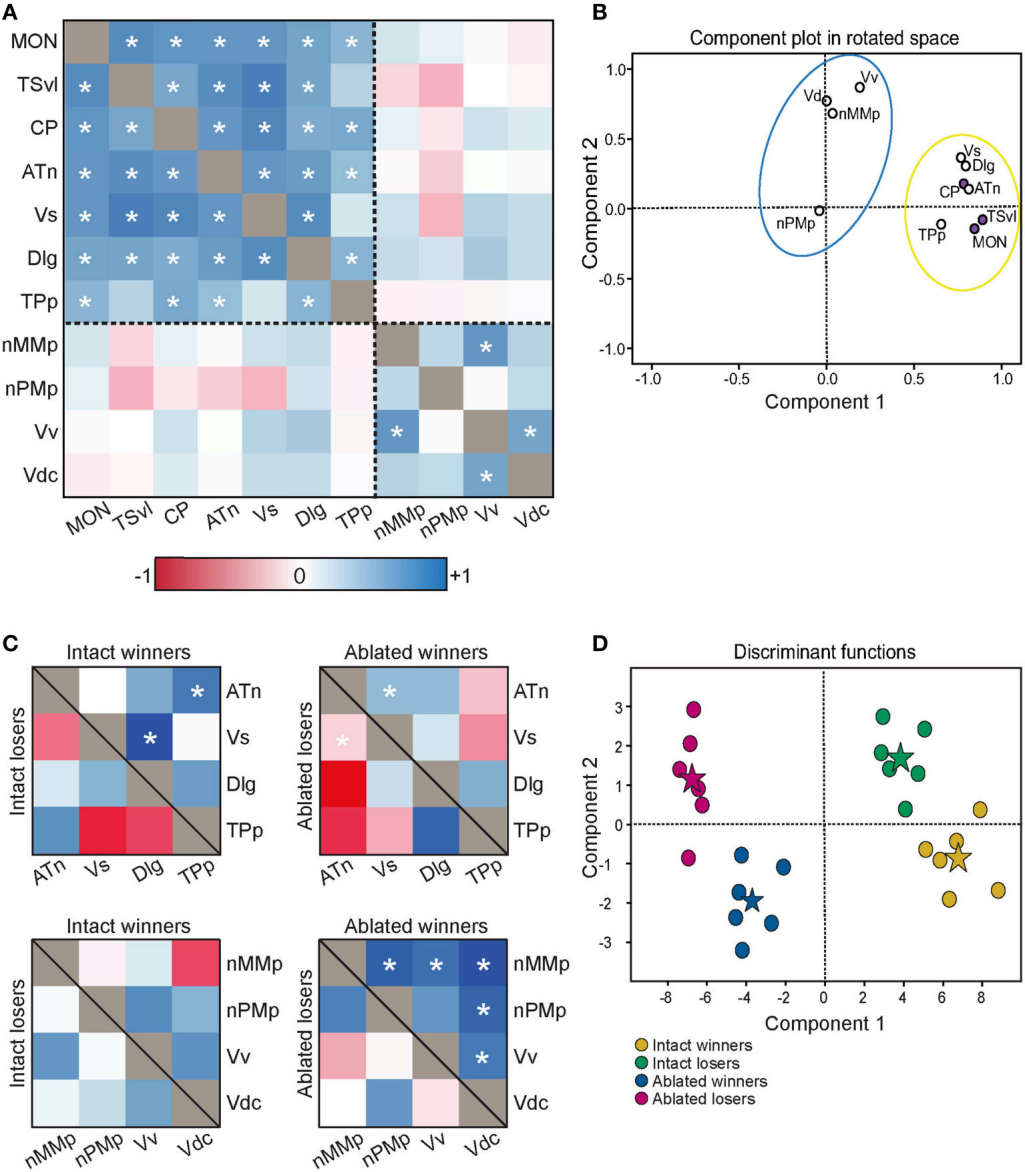
**Lateral line-ablated animals have altered co-activation of functional networks within the SDMN. (A)** Heat map of Pearson correlation coefficients (*R* = color scale) of *cfos* staining in brain nuclei correlated with other nuclei (*N* = 24 animals per region; See also Table 2). Dashed lines separate the two functional networks. **(B)** Principal component analysis of *cfos* staining in sensory processing (purple dots) and social behavior (white dots) brain nuclei reveal two distinct networks. Dashed circle encompasses network that incorporates mechanosensory cues, and solid circle encompasses second network without lateral line input. **(C)** Heat maps of Pearson correlation coefficients of two functional networks within the SDMN (*N* = 6 animals per group). Top two boxes represent network 1 (SDMN nuclei that receive lateral line input) with winners above the black line and losers below. Bottom two boxes represent network 2 (other SDMN nuclei). **(D)** Discriminant function analysis distinguished lateral line-intact fight winners (yellow) and losers (green) from lateral line-ablated fight winners (blue) and losers (pink). Stars represent each group centroid. ^*^P < 0.05 on heat maps. See list for abbreviations.

To determine the functional connectivity of the nodes of the SDMN, we first correlated *cfos* staining across brain regions (Figure 7A, Table 2). Lateral line processing regions (MON, TSvl, and CP) all positively correlated with each other. In addition, the ATn, Vs, and Dlg positively correlated with each other and with lateral line processing regions, providing further evidence that they process mechanosensory information. *Cfos* staining in the Vv positively correlated with the nMMp and the Vdc. There were no correlations between lateral line processing regions and the nMMp, nPMp, Vv, or Vdc suggesting that activation of these regions is not mediated by mechanosensory input in this agonistic context. Principal component analysis (PCA) of *cfos* activation in lateral line processing and SDMN nuclei produced two significant components describing the variability in the data (Figure 7B; Table 3; *N* = 24 animals; Kaiser-Meyer-Olkin measure of sampling adequacy = 0.505; Bartlett's test of sphericity chi-squared = 122.741, *df* = 66, *P* < 0.001). The first component (42.489% of variance) was strongly weighted by the MON, TSvl and ATn, and the second component (17.533% of variance explained) was weighted primarily by the Vv and nMMp. Based on the nodes driving each component, component 1 most likely represents mechanosensory input and component 2 likely represents social behavior regions relating to reproduction and social status. Based on these correlations and PCA, we identified two distinct functional networks within the SDMN. The first consists of the ATn, Vs, Dlg, and TPp, and the second consists of the Vv, Vd, nMMp, and nPMp. In the context of this territorial behavioral paradigm, these two functional networks likely represent two distinct networks within the SDMN: one that receives mechanosensory signals to mediate behavioral output, and the other that acts independent of mechanosensory input.

**Table 2:** **Pearson correlation values of *cfos* staining in each brain nucleus correlated with all other brain nuclei**.

		**TSvl**	**CP**	**ATn**	**Vs**	**Dlg**	**TPp**	**nMMp**	**nPMp**	**Vv**	**Vdc**	**TSc**	**Dp**	**Dc-4**
MON	R	**0.694**	**0.650**	**0.644**	**0.647**	**0.573**	**0.476**	0.181	0.122	−0.031	−0.074	0.237	**0.682**	**−0.524**
	P	**<0.001**	**<0.001**	**<0.001**	**<0.001**	**0.006**	**0.019**	0.408	0.578	0.886	0.738	0.265	**<0.001**	**0.012**
TSvl	R		**0.554**	**0.686**	**0.765**	**0.563**	0.305	−0.149	−0.277	0.010	−0.043	0.184	**0.789**	**−0.676**
	P		**0.006**	**<0.001**	**<0.001**	**0.010**	0.158	0.508	0.211	0.963	0.850	0.401	**<0.001**	**<0.001**
CP	R			**0.634**	**0.778**	**0.528**	**0.552**	0.103	−0.093	0.215	0.147	0.110	**0.528**	−0.350
	P			**<0.001**	**<0.001**	**0.014**	**0.005**	0.639	0.672	0.314	0.503	0.610	**0.014**	0.110
ATn	R				**0.647**	**0.609**	**0.440**	−0.028	−0.176	0.020	0.037	0.132	**0.581**	−0.339
	P				**<0.001**	**0.003**	**0.031**	0.898	0.421	0.923	0.867	0.540	**0.006**	0.123
Vs	R					**0.696**	0.177	0.217	−0.268	0.309	0.245	0.305	**0.817**	−0.423
	P					**<0.001**	0.418	0.331	0.228	0.151	0.273	0.157	**<0.001**	0.056
Dlg	R						**0.487**	0.246	0.190	0.356	0.244	0.349	**0.757**	−0.149
	P						**0.025**	0.217	0.410	0.113	0.287	0.120	**<0.001**	0.543
TPp	R							−0.044	−0.053	−0.036	0.020	0.040	9.279	−0.174
	P							0.842	0.811	0.867	0.929	0.852	0.220	0.439
nMMp	R								0.267	**0.636**	0.304	**0.438**	0.100	0.144
	P								0.217	**0.001**	0.170	**0.037**	0.673	0.534
nPMp	R									0.028	0.261	0.003	−0.216	0.232
	P									0.901	0.241	0.987	0.361	0.312
Vv	R										**0.563**	**0.566**	0.215	0.415
	P										**0.005**	**0.004**	0.349	0.055
Vdc	R											0.153	0.195	0.385
	P											0.485	0.398	0.085
TSc	R												0.310	0.328
	P												0.171	0.136
Dp	R													**−0.555**
	P													**0.014**

*Pearson correlation values (R) were used to create the heat map in Figure 7A. Bold indicates significance at P < 0.050*.

**Table 3:** **Loading values for multivariate analyses**.

**PCA (Figure** **7B****) component loadings**			
	**Component 1**	**Component 2**	**Component 3**
MON	**0.770**	−0.318	0.370
TSvl	**0.842**	−0.329	−0.168
CP	**0.812**	−0.053	−0.040
ATn	**0.829**	−0.089	−0.058
Vs	**0.851**	0.118	−0.229
Dlg	**0.826**	0.106	0.217
Vv	0.425	**0.780**	−0.237
Vdc	0.201	**0.719**	−0.090
TPp	0.593	−0.268	0.106
nMMp	0.205	**0.690**	0.361
nPMp	−0.072	0.096	**0.921**
**CDA (Figure** **7D****) factor loadings**			
	**Factor 1**	**Factor 2**	**Factor 3**
MON	0.183	**0.376**	0.071
TSvl	**0.419**	**0.425**	0.185
CP	0.132	0.073	−0.072
ATn	0.168	**0.314**	**−0.340**
Vs	**0.208**	0.118	−0.011
Dlg	0.151	0.103	−0.124
Vv	0.010	**−0.207**	−0.017
Vdc	0.013	**−0.239**	**−0.261**
TPp	0.072	−0.059	0.129
nMMp	0.008	−0.112	0.142
nPMp	−0.081	**0.277**	0.044
**PCA (Figure** **8C****) component loading**			
	**Component 1**	**Component 2**	**Component 3**
MON	**0.793**	–	–
TSvl	**0.869**	–	–
ATn	**0.806**	–	–
TPp	**0.566**	–	–
CP	**0.818**	–	–
VS	**0.848**	–	–
nMMp	–	**0.834**	–
nPMp	–	–	0.398
Dlg	**0.791**	–	–
Vv	0.323	**0.657**	0.315
Vdc	–	0.346	**0.709**
BehaviorChoice	**−0.674**	–	0.483
Aggression	–	0.470	**−0.690**
Latency	–	−0.396	–
Fight	0.460	–	0.384
Assessment	**−0.526**	–	0.419
Pre-fight	0.553	0.556	–

*Component loadings for principle component analyses and discriminant function analysis. Bold indicates the highest loading factors (>0.5 for PCA and >0.2 for CDA)*.

To compare activation and connectivity of these functional networks in lateral line-intact and -ablated fight winners and losers, we used Pearson correlations and created heat maps based on the correlation coefficients (Figure 7C). While few significant correlations were detected (likely due to small sample sizes; *N* = 6 for each group), heat maps allow for better visualization of the differences. The most striking difference occurs in lateral line-ablated fight winners. No other group had significant correlations (i.e., co-activity) of the functional network containing the Vv, Vd, nMMp, and nPMp; however, lateral line-ablated fight winners had strong co-activity of all regions within this network and little co-activity within the functional network containing mechanosensory-mediated brain nuclei. These data indicate that in the absence of mechanosensory input, activation of functional networks within the SDMN is altered.

To further examine these differences in network activation, we next used canonical discriminant function analysis (CDA; Figure 7D; Table 3). Discriminant function analysis combines all input variables into a single composite score and can identify which variables (i.e., brain regions) contribute to differentiation between the four animal groups. CDA verifies that animals can be distinguished via brain activation alone and can provide insight into which nodes of the SDMN are driving these distinctions. Our discriminant function analysis accurately predicted 100% of ablated winners, 83.33% of ablated losers, but only 50% of lateral line-intact fish, and there was no cross over between lateral line-intact and -ablated animals. Furthermore, all of these values exceed classification by chance (25%). Component 1 was driven by the MON, TSvl, and the Vs, and explained 84.6% of the variance in the data. Component 2 was loaded primarily by the TSvl, MON, nMMp, nPMp, ATn, Vd, and Vv, and only explained 14% of the data, and component 3 was equally loaded by all brain regions and only accounted for <2% of the variance. Together, the first 2 components explained more than 98% of the variance in the data and clearly separated the four animal groups based on brain activation alone.

### Correlation of behavior and brain activation data

**Figure 8:**
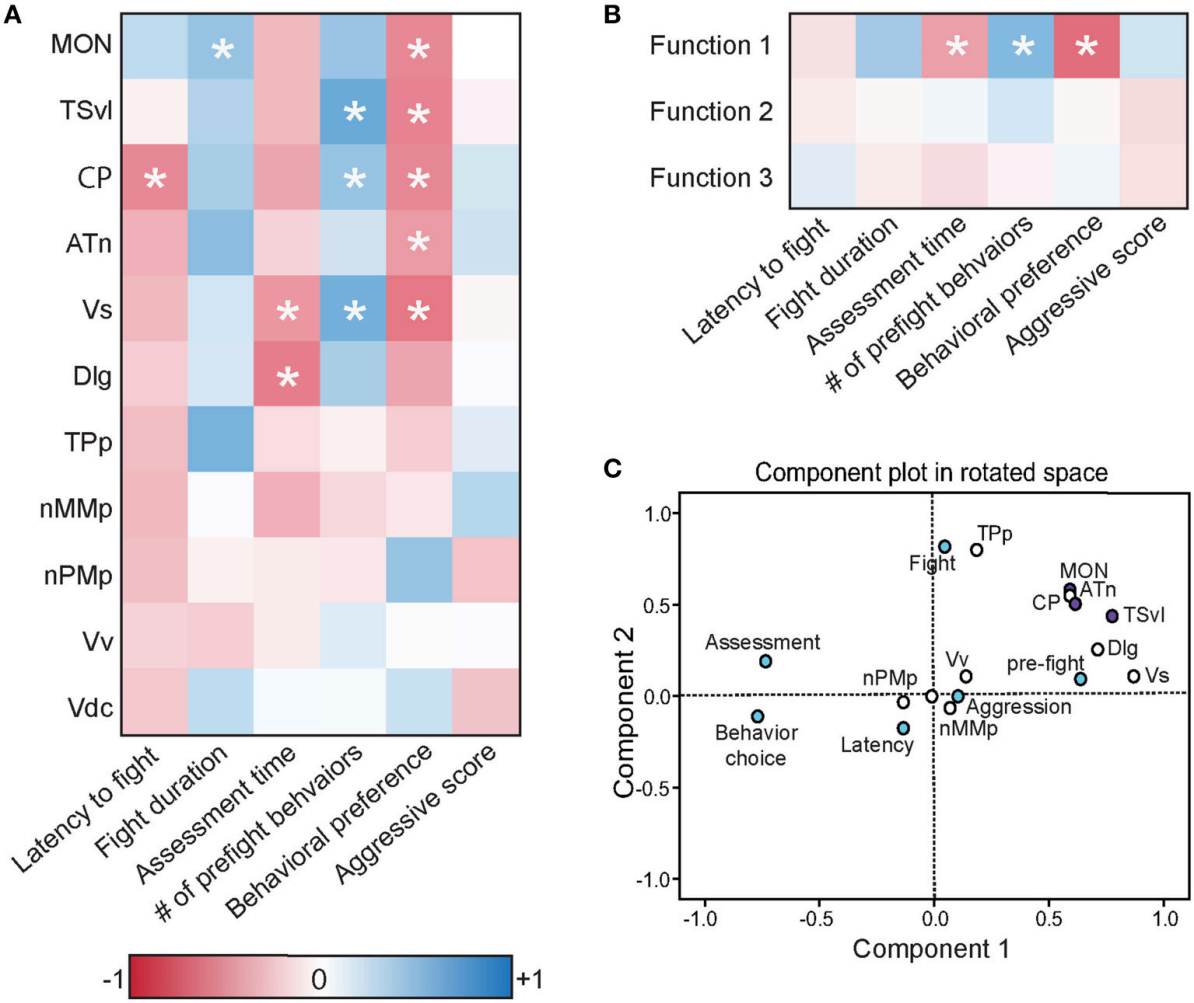
**Brain *cfos* staining correlates with fight and assessment behaviors. (A)** Heat map of Pearson correlation coefficients (*R* = color scale) of *cfos* staining in brain nuclei with behaviors (see also Table 4). **(B)** Heat map of Pearson correlation coefficients of the CDA functions with behaviors. Function 1 (loaded primarily by the MON, TSvl, ATn, Dlg, and Vs) separated lateral line-intact and -ablated animals. Function 2 (loaded primarily by the TSvl, MON, nMMp, nPMp, ATn, Vdc, and Vv) separated fight winners and losers. Function 3 was equally loaded by all brain regions. **(C)** Principal component analysis of *cfos* staining in sensory processing regions (purple dots), social behaviors (white dots), and fight and assessment behaviors (blue dots). ^*^P < 0.05. See list for abbreviations. Aggression: aggressive score during territorial interaction. Assessment: % of prefight time spent within one body length of opponent. Behavior choice: ratio of contact to non-contact fight behaviors. Fight: fight duration. Latency: latency to fight. Pre-fight: number of unreciprocated prefight aggressive behaviors.

To further understand the role of the SDMN in processing socially-relevant mechanosensory signals, we correlated fight behaviors with brain *cfos* activation data (Figure 8A; Table 4). Latency of fight time negatively correlated only with *cfos* activation in the CP (*R* = −0.497, *P* = 0.014), while fight duration positively correlated with the MON (*R* = 0.404, *P* = 0.047). Assessment time negatively correlated with the number of *cfos*-labeled cells in both the Vs (*R* = −0.432, *P* = 0.039) and the Dlg (*R* = −0.537, *P* = 0.012) and the number of pre-fight behaviors positively correlated with the TSvl, CP, and Vs (*R* > 0.40, *P* < 0.05 for each). In addition, behavioral preference (ratio of contact to non-contact behaviors) negatively correlated with *cfos* activation in all three lateral line processing regions, the ATn, and the Vs (*R* < −0.40, *P* < 0.05 for each), but aggressive scores did not correlate with any quantified regions. The nMMp, nPMp, Vv, and Vd did not correlate with any fight behaviors. We also correlated the three discriminant function scores of each animal to their behaviors (Figure 8B). Function 1 (loaded primarily by mechanosensory-mediated brain regions) negatively correlated with behavioral preference (*R* = −0.618, *P* < 0.001) and assessment time (*R* = −0.406, *P* = 0.049), and positively correlated with the number of pre-fight aggressive behaviors (*R* = 0.478, *P* = 0.018). Functions 2 and 3, however, did not correlate with any measured fight behaviors.

**Table 4:** **Correlations of fight and assessment behaviors with brain *cfos* quantification data**.

		**Latency to fight**	**Fight duration**	**Time within 1 body length**	**Number of pre-fight behaviors**	**C:NC behavior ratio**	**Aggressive score**
MON	R	−0.243	**0.404**	−0.289	0.396	**−0.502**	0.002
	P	0.254	**0.047**	0.170	0.055	**0.012**	0.993
TSvl	R	−0.053	0.300	−0.288	**0.578**	**−0.518**	−0.045
	P	0.811	0.056	0.182	**0.004**	**0.011**	0.829
CP	R	**−0.497**	0.338	−0.374	**0.405**	**−0.502**	0.172
	P	**0.014**	0.296	0.072	**0.050**	**0.012**	0.420
ATn	R	−0.330	0.461	−0.176	0.179	**−0.425**	0.182
	P	0.115	0.095	0.411	0.404	**0.039**	0.395
Vs	R	−0.297	0.174	**−0.432**	**0.547**	**−0.581**	−0.030
	P	0.168	0.650	**0.039**	**0.007**	**0.003**	0.892
Dlg	R	−0.203	0.153	**−0.537**	0.331	−0.377	0.023
	P	0.377	0.507	**0.012**	0.142	0.092	0.920
TPp	R	−0.28	0.536	−0.143	−0.063	−0.204	0.113
	P	0.185	0.055	0.505	0.769	0.340	0.598
nMMp	R	−0.29	0.022	−0.331	−0.156	−0.093	0.283
	P	0.180	0.950	0.123	0.476	0.672	0.191
nPMp	R	−0.270	−0.054	−0.086	−0.094	0.404	−0.257
	P	0.213	0.730	0.697	0.671	0.056	0.237
Vv	R	−0.193	−0.204	−0.086	0.125	−0.009	0.015
	P	0.366	0.816	0.690	0.560	0.966	0.946
Vdc	R	−0.231	0.241	0.024	0.037	0.201	−0.250
	P	0.289	0.268	0.914	0.869	0.358	0.249
F1	R	−0.124	0.353	**−0.406**	**0.478**	**−0.618**	0.185
	P	0.563	0.091	**0.049**	**0.018**	**<0.001**	0.388
F2	R	−0.073	−0.030	0.045	0.166	−0.030	−0.149
	P	0.734	0.889	0.835	0.439	0.889	0.488
F3	R	0.115	−0.073	−0.136	−0.046	0.062	−0.127
	P	0.592	0.734	0.525	0.833	0.775	0.555

*Pearson correlation values (R) of cfos quantification data in brain nuclei correlated with behaviors. Data used to create heat maps in Figures 8A,C. F1, F2, and F3 are discriminant scores of each animal for functions 1, 2, and 3. Bold indicates significance at P < 0.050*.

To further examine the relationship between fight behaviors and activation of sensory and socially-relevant brain regions, we ran a PCA on brain and behavior data together (Figure 8C; Table 3). This analysis pulled out three main components (*N* = 24 animals; Kaiser-Meyer-Olkin measure of sampling adequacy = 0.301; Bartlett's test of sphericity chi-squared = 207.794, *df* = 136, *P* < 0.001). The first consisting primarily of lateral line processing regions, along with their correlated behaviors (behavioral choice, assessment abilities, and number of pre-fight behaviors), and this component explained 34.224% of variance. Component 2 was loaded primarily by the TPp and fight duration (13.558% variance explained), and component 3 was loaded by the nMMp, nPMp Vv, Vdc, aggressive score, and latency to fight (10.959% variance explained). The correlation of assessment behaviors and behavioral choice with regions of the SDMN and functional network co-activity further indicates the crucial role of mechanosensory cues in mediating these behaviors.

## Discussion

**Figure 9:**
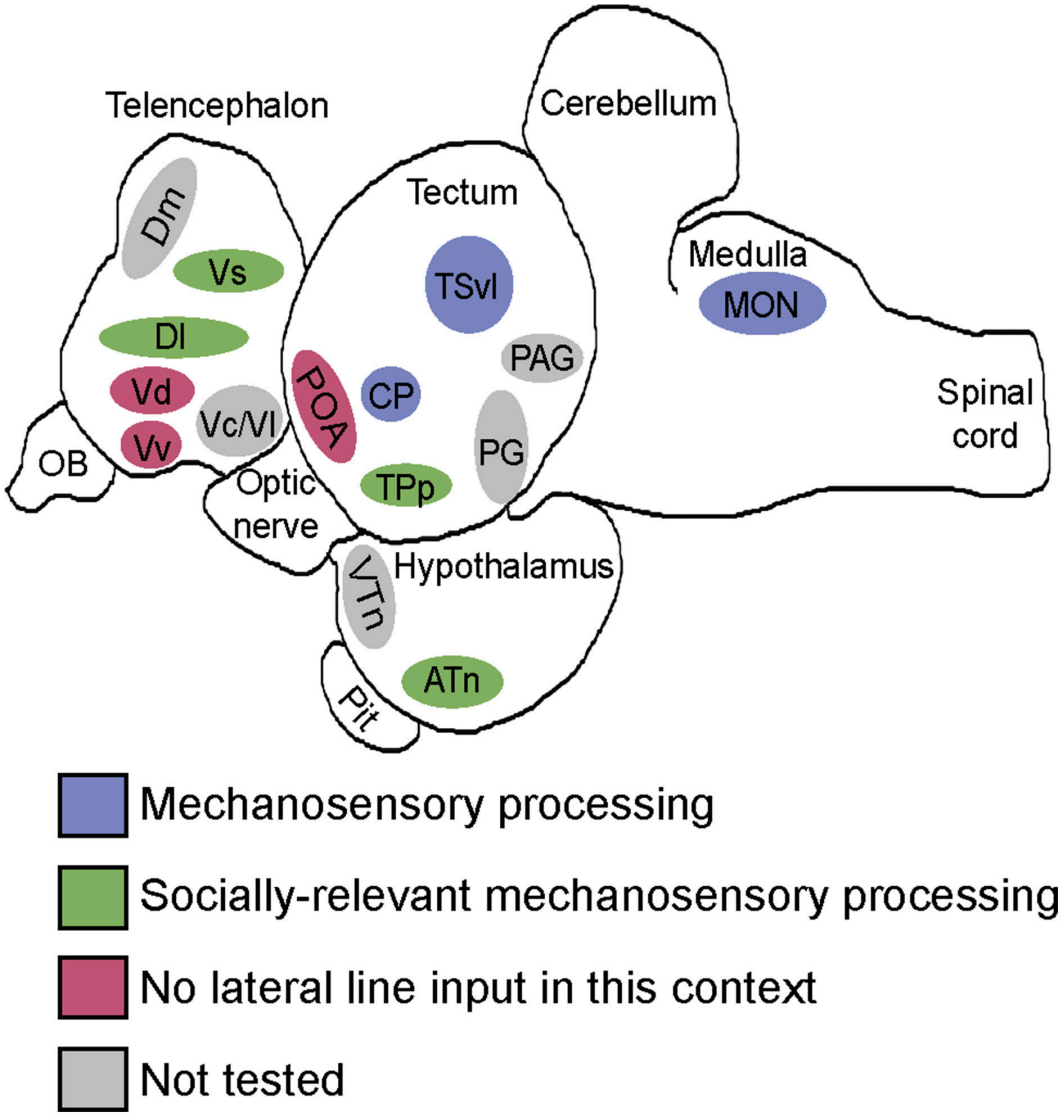
**Summary schematic of socially-relevant mechanosensory processing in the cichlid brain**. Blue represents mechanosensory processing regions. Green represents nuclei of the SDMN that receive lateral line input and possibly use it to modify behavioral output. Pink represents SDMN nuclei without evidence for mechanosensory input during aggressive contexts. Gray represents SDMN and sensory processing regions not tested for a potential role in processing socially-relevant mechanosensory signals. Locations of nuclei on the sagittal brain outline are only approximate. See list for abbreviations.

We provide for the first time in any fish species evidence of where socially-relevant mechanosensory information is processed in the brain (Figure 9). After identifying nuclei that receive and process mechanosensory signals, we used a network activity approach to identify two distinct functional networks. During male-male territorial interactions, one network consisting of the ATn, Vs, Dlg, and TPp receives and processes mechanosensory signals to modify behavioral output while the second network consisting of the Vv, Vdc, nMMp, and nPMp acts independent of mechanosensory input. These data provide novel insight into how mechanosensory cues are integrated into brain networks controlling social behaviors to produce context-appropriate behavioral responses.

### Impact of cobalt chloride treatment on sensory processing

We first examined *cfos* staining in sensory processing brain nuclei of lateral line-ablated and -intact fight winners and losers to verify that cobalt chloride impaired reception of mechanosensory cues. While previous studies have used neural tract-tracing and electrophysiological recording techniques in anesthetized fish to elucidate some of the lateral line processing regions in the brain (reviewed in Wullimann and Grothe, 2014), our work is the first to identify mechanosensory processing regions in naturally behaving animals with and without lateral line input. Lateral line-ablated fish had decreased *cfos* staining (indicating decreased activation) in all examined lateral line processing regions (MON, TSvl, CP). Treatment efficacy was initially verified via reduced DASPEI staining of neuromasts (Butler and Maruska, 2015), and reduced *cfos* staining in lateral line brain regions further demonstrates that our CoCl_2_ treatment functionally disabled neuromasts of the mechanosensory lateral line system. CoCl_2_ treatment reduced evoked activity in the posterior lateral line nerve of the roach *Rutilus rutilus* (Karlsen and Sand, 1987), but there is a paucity of studies verifying CoCl_2_ treatment efficacy beyond neuromast vital dye staining. All lateral line-ablated animals had some positive *cfos* staining in lateral line processing regions, suggesting that ablation may not have been 100%. It is possible that the observed *cfos* staining in lateral line-ablated animals was due to remaining functional neuromasts, spontaneous activity in the anterior lateral line nerves, or activation via circuits from other nuclei.

We also examined *cfos* staining in central processing regions of auditory and olfactory signals to look for comorbid effects on other sensory systems. Karlsen and Sand (1987) previously showed that cobalt treatment had no impact on hearing capabilities (measured via utricular microphonic potentials) in *R. rutilus*, but because of the higher dose used in our experiments and the species variability of CoCl_2_ treatments, we also quantified *cfos* staining in the auditory TSc. We found no difference between lateral line-intact and -ablated animals in the TSc, indicating that our treatment had no impact on hair cells of the inner ear. We previously found that fish treated with 2 mM CoCl_2_ had noticeably reduced staining in the olfactory epithelium (Butler and Maruska, 2015), suggesting an impact on olfaction. In addition, CoCl_2_ treated animals had decreased *cfos* staining in olfactory processing regions (e.g., olfactory bulb and Dp) indicating that cobalt chloride also impairs sensory cells of the olfactory epithelium in addition to neuromasts of the lateral line system (and although not tested, cobalt likely also impacts taste buds and solitary chemosensory cells located on the surface of fish). *A. burtoni*, like many teleost fishes, use multimodal signaling during social behaviors, and males use chemosensory signaling during male-male social interactions (Maruska and Fernald, 2012). Because of this, we included anosmic (olfactory epithelium ablated) animals in our study and compared behavior and brain activation of anosmic winners to lateral line-intact winners. These data and controls allowed us to be confident that any observed behavioral and *cfos* expression differences were due solely to loss of mechanoreceptive capabilities and not olfaction.

### Central processing of socially-relevant mechanosensory cues

The social decision making-network was first proposed as an integrative network incorporating the highly connected SBN and mesolimbic reward system (O'Connell and Hofmann, 2011). Together, these networks are thought to be involved in mediating adaptive (i.e., rewarding) social behaviors by evaluating the salience of a sensory cue and integrating it with the animals own internal physiology to produce an appropriate behavioral response. We first determined which nuclei of the SDMN receive mechanosensory cues by examining *cfos* staining differences in each brain region, and then examined how co-activation of functional networks within the SDMN are influenced by mechanosensory cues. The ATn receives direct and indirect input from the torus semicircularis (TS) and is considered by some to be an octavolateralis nucleus because of these connections (Striedter, 1991; Wullimann and Grothe, 2014). It is, therefore, an ideal candidate for linking mechanosensory information with other brain nuclei implicated in social decision-making. Although the ventromedial hypothalamus (VMH; mammalian homolog of the ATn) plays an important role in mediating aggressive and reproductive behaviors in mammals (Olivier, 1977; Kollack-Walker and Newman, 1995; Lin et al., 2011; Yang et al., 2013) there is little evidence of the functional role of the ATn in teleost fishes. The ATn expresses sex-steroid receptors (Munchrath and Hofmann, 2010), is activated following social encounters, and in *A. burtoni*, is likely involved in social status transitions (Maruska et al., 2013). In *A. burtoni*, the ATn processes socially-relevant mechanosensory cues, as indicated by the decreased *cfos* staining in the ATn of lateral line-ablated animals and the negative correlation of *cfos* staining in the ATn with behavioral choice (contact vs. non-contact behaviors). It is possible, therefore, that mechanosensory cues could influence behavioral choice, social interactions, and social status transitions via the ATn and its connections.

The ATn and other lateral line processing regions project directly to several forebrain nuclei of the SDMN, such as the Dm, Dl, and Dc (Yamamoto and Ito, 2008; Wullimann and Grothe, 2014). Lateral line-ablated animals had reduced staining in the Vs and Dlg compared to lateral line-intact fish indicating that both regions receive hydrodynamic input. In addition, both regions negatively correlated with assessment time, and *cfos* staining in the Vs positively correlated with the number of unreciprocated pre-fight aggressive behaviors and negatively with the animal's behavioral preference. These correlations suggest that not only do these regions receive lateral line input, but they may also be involved in animal assessment and behavioral choices. Several tract-tracing studies have demonstrated connections between mechanosensory processing regions in pallial brain nuclei (Dl, Dc, Dm), but this is the first evidence of a subpallial nucleus (Vs) receiving mechanosensory information. The Vs is thought to be homologous in part to the mammalian medial extended amygdala (O'Connell and Hofmann, 2011), which is known for its role in incorporating sensory cues (Turner and Herkenham, 1991; Gray, 1999) to modify behaviors, and it is possible that the Vs has a similar role in the teleost brain.

### Co-activation of functional networks of the SDMN depends on mechanosensory input

When first describing the SBN, Newman proposed that specific behavioral states (e.g., male sexual behavior) are most likely due to the pattern of activation across nodes of the SBN rather than at one specific node (Newman, 1999; Yang and Wilczynski, 2007; Teles et al., 2015). To examine this, we first sought to determine the functional networks within the SDMN in the context of socially-relevant hydrodynamic cues. Correlation heat maps and PCA identified two distinct networks. The first consisted of lateral line sensory processing regions, regions of the SDMN that receive lateral line input, and the TPp. This network was primarily loaded by the MON, TSvl, CP, and ATn indicating that it is dependent on lateral line input. The inclusion of SDMN regions (ATn, Vs, Dlg, and TPp) with sensory processing regions (MON, TSvl, CP) in this network provides further evidence for mechanosensory input to these nuclei. The second network consisted of the remaining SDMN nuclei (Vv, Vdc, nMMp, and nPMp). These nuclei have functions related to reproduction and social status, and activation of these regions appears to be independent of mechanosensory input. In the context of male-male agonistic interactions, it is possible that the first component (or network) is closely tied to the fight itself while the second network is more closely related to winning an agonistic interaction and acquiring a spawning territory (i.e., reproductive fitness and status position consequences). Analyses of co-activity of these functional networks in lateral line-intact and -ablated fight winners and losers revealed distinct activation patterns. For example, lateral line-intact fight winners had significant, positive co-activity of the network containing the Vs, Dlg, ATn, and TPp and no significant co-activity of the second network, but lateral line-ablated fight winners had little co-activation of the first network and strong co-activation of the second. One hypothesis is that the first network (receiving lateral line input) is dominating the output of the SDMN when mechanoreceptive capabilities are intact, but without mechanosensory input, activation of the SDMN is driven more by the fight outcome itself (i.e., acquiring a spawning territory). These differences in connectivity and activation across functional networks indicate that socially-relevant hydrodynamic cues are essential to the SDMN.

The role of interconnected neural networks rather than individual nuclei is likely also applicable to complex social behaviors such as opponent assessment. Animal assessment is extensively studied via behavioral analyses (reviewed in Arnott and Elwood, 2009), but little is known about which brain regions are involved in opponent mutual assessment. Because mutual assessment is dependent on integration of multimodal sensory information, it is likely that assessment behaviors are dependent on a network of nuclei working together to integrate sensory cues with the animal's own internal physiology. Our data indicate that during male-male territorial disputes, assessment abilities correlate the co-activity of mechanosensory processing regions and the ATn, Vs, Dlg, and TPp. This novel result further supports the notion that mechanosensory cues are used to assess opponents and provides the first evidence for where in the teleost brain this mutual assessment may occur.

## Conclusions

Here we show for the first time that socially-relevant hydrodynamic cues are processed in nodes of the SDMN and that without lateral line input, fish have altered activation of functional networks within the SDMN. Furthermore, lateral line-ablated fish alter their behavioral choices and have decreased assessment ability, which is related to activation of these functional networks in the brain. Although the role of visual, chemosensory, and auditory cues are well-studied for their role(s) in social behaviors, our data indicates that the mechanosensory lateral line system is an integral part of social interactions and mediates activation of brain regions used in social decisions. Given that many social interactions among the >30,000 different species of fishes produce hydrodynamic cues that can be detected by the lateral line system, a better understanding of how this mechanosensory information is integrated in the brain to produce context-dependent behaviors also has broad implications for the evolution of SDMN function across vertebrates.

## Author contributions

Both authors had full access to the data and take full responsibility for the integrity of the data analysis and approved the final manuscript. JB and KM designed experiments. JB performed experiments and analyzed data. JB and KM wrote the manuscript. KM provided funding, equipment, and supplies.

## Funding

Funding was provided by startup funds from the College of Science and Department of Biological Sciences at Louisiana State University (KM), Louisiana Board of Regents RCS Grant (KM), Powe Faculty Enhancement Award from ORAU (KM), Sigma Xi (JB), and a Raney Award from ASIH (JB). JB was supported by a Louisiana Board of Regents Graduate Fellowship and National Science Foundation Graduate Research Fellowship.

### Conflict of interest statement

The authors declare that the research was conducted in the absence of any commercial or financial relationships that could be construed as a potential conflict of interest.

## Abbreviations

ATn: anterior tuberal nucleus
ANOVA: analysis of variance
BM: body mass
CC: cerebellar crest
CDA: canonical discriminant function analysis
CoCl_2_: cobalt chloride
CP: central posterior thalamic nucleus
DASPEI: 2-[4-(dimethylamino)styryl]-*N*-ethylpyridinium iodide
Dc: central part of the dorsal telencephalon
Dc-4: Dc, subdivision 4
Dl: lateral zone of the dorsal telencephalon
Dlg: granular zone of Dl
Dm: medial part of the dorsal telencephalon
Dp: posterior portion of the dorsal telencephalon
E: entopeduncular nucleus
EGTA: ethylene glycolbis tetraacetic acid
GSI: gonadosomatic index
ICL: inner cellular layer of the OB
MON: medial octavolateralis nucleus
NLT: lateral tuberal nucleus
nMMp: magnocellular preoptic nucleus, magnocellular division
nPMp: magnocellular preoptic nucleus, parvocellular division
OB: olfactory bulb
PAG: periaqueductal gray
PCA: principal component analysis
Pit: pituitary
PGl: lateral preglomerular nucleus
PTT: paratoral tegmental nucleus
SBN: social behavior network
SDMN: social decision-making network
SL: standard length
TPp: periventricular nucleus of the posterior tuberculum
TSc: central portion of the torus Semicircularis
TSvl: ventrolateral portion of the torus Semicircularis
Vc: central part of the ventral telencephalon
VCeg: granular layer of vulvula cerebellum
Vd: dorsal part of the ventral telencephalon
Vdc: caudal subdivision of Vd
Vl: lateral zone of the ventral telencephalon
Vp: postcommissural nucleus of the ventral telencephalon
Vs: supracommissural nucleus of the ventral telencephalon
VTn: ventral tuberal nucleus
Vv: ventral part of the ventral telencephalon.
